# β-Arrestin Regulates Estradiol Membrane-Initiated Signaling in Hypothalamic Neurons

**DOI:** 10.1371/journal.pone.0120530

**Published:** 2015-03-24

**Authors:** Angela M. Wong, Matthew C. Abrams, Paul E. Micevych

**Affiliations:** Department of Neurobiology David Geffen School of Medicine at UCLA and Laboratory of Neuroendocrinology of the Brain Research Institute, at University of California Los Angeles, Los Angeles, California, United States of America; University of Naples 2, ITALY

## Abstract

Estradiol (E2) action in the nervous system is the result of both direct nuclear and membrane-initiated signaling (EMS). E2 regulates membrane estrogen receptor-α (ERα) levels through opposing mechanisms of EMS-mediated trafficking and internalization. While ß-arrestin-mediated mERα internalization has been described in the cortex, a role of ß-arrestin in EMS, which underlies multiple physiological processes, remains undefined. In the arcuate nucleus of the hypothalamus (ARH), membrane-initiated E2 signaling modulates lordosis behavior, a measure of female sexually receptivity. To better understand EMS and regulation of ERα membrane levels, we examined the role of ß-arrestin, a molecule associated with internalization following agonist stimulation. In the present study, we used an immortalized neuronal cell line derived from embryonic hypothalamic neurons, the N-38 line, to examine whether ß-arrestins mediate internalization of mERα. β-arrestin-1 (Arrb1) was found in the ARH and in N-38 neurons. *In vitro*, E2 increased trafficking and internalization of full-length ERα and ERαΔ4, an alternatively spliced isoform of ERα, which predominates in the membrane. Treatment with E2 also increased phosphorylation of extracellular-signal regulated kinases 1/2 (ERK1/2) in N-38 neurons. Arrb1 siRNA knockdown prevented E2-induced ERαΔ4 internalization and ERK1/2 phosphorylation. *In vivo*, microinfusions of Arrb1 antisense oligodeoxynucleotides (ODN) into female rat ARH knocked down Arrb1 and prevented estradiol benzoate-induced lordosis behavior compared with nonsense scrambled ODN (lordosis quotient: 3 ± 2.1 vs. 85.0 ± 6.0; p < 0.0001). These results indicate a role for Arrb1 in both EMS and internalization of mERα, which are required for the E2-induction of female sexual receptivity.

## Introduction

Estrogens act at cell membrane receptors to activate intracellular signaling, which is implicated in many brain functions including the regulation of female sexual receptivity [1, 2]. In estradiol (E2) membrane-initiated signaling (EMS), estrogen receptor-α (ERα) transactivates metabotropic glutamate receptors (mGluRs; [3–5]) regulating signaling pathways [6–12]. Plasma membrane ER (mER) levels are determined by a balance of trafficking to the membrane, requiring ER palmitoylation and interaction with caveolin-1 (CAV1; [13, 14]), and internalization, requiring β-arrestin-1 (Arrb1; [15]). To study EMS, we used previously characterized immortalized hypothalamic neurons (N-38s). These cells expressed neuropeptide Y, full length ERα and ERαΔ4, a splice variant lacking exon 4. ERαΔ4 is enriched in the plasma membranes of cultured neurons and astrocytes, and its mRNA is widely distributed in the CNS [16–19]. Trafficking of ERα and ERαΔ4 to the membrane and internalization is regulated in parallel by E2 [17, 18]. As in other neuronal hypothalamic cells that have membrane ERs, N-38 neurons respond to E2 treatment by increasing free cytoplasmic calcium levels ([Ca^2+^]_i_), and by activating extracellular signal-regulated kinases 1/2 and protein kinase C (PKC).

Internalization is an important aspect of membrane receptor dynamics and limits cellular responses initiated by agonist stimulation of G protein-coupled receptors (GPCRs). Receptor stimulation leads to GPCR kinase (GRK) activation, which results in the phosphorylation of receptors including mERα [15, 20]. With other GPCRs, β-arrestins bind to phosphorylated receptors, uncouple G proteins and link receptors to clathrin-dependent internalization pathways [21–24]. A more recently discovered function of β-arrestins is to organize members of the ERK1/2 (aka mitogen-activated protein kinase, MAPK) pathway [24–27]. While it is unknown whether β-arrestins are involved in organizing signaling molecules for mERs, E2 activates ERK1/2 [3, 28], potentially through a β-arrestin-mediated mechanism. Thus, β-arrestins may be crucial not only for limiting E2 signaling via mERα internalization, but may be involved in the initial EMS through an ERK1/2 pathway. This rapid, membrane-initiated action of E2 is important for activating the limbic-hypothalamic lordosis-regulating circuit, in which stimulating β-endorphin release activates μ-opioid receptors (MOR) in the medial preoptic nucleus ([29], reviewed in [30, 31]).

Within this lordosis-regulating circuit, E2 activates NPY-expressing neurons in the ARH, which we modeled with NPY mRNA expressing N-38 neurons in the present studies. Following E2 activation of N-38 neurons, calcium levels increase [17] and ERK1/2 is phosphorylated [15]. *In vivo*, EMS activates a transiently inhibitory circuit that is ultimately necessary for the full display of lordosis behavior [2, 29, 32].

We hypothesize that Arrb1 knockdown would abrogate EMS and consequently lordosis behavior. Thus, in this study, we examined the role of Arrb1 regulation of E2-induced ER internalization and subsequent ERK1/2 signaling in N-38 neurons. Arrb1 siRNA was used to reduce Arrb1 protein levels. In addition, we tested whether Arrb1 knockdown *in vivo* regulated sexual receptivity using Arrb1 antisense oligodeoxynucleotides (asODN) infused into the ARH prior to estradiol benzoate (EB) priming.

## Materials and Methods

### N-38 cultures

N-38 neurons were obtained from CELLutions Biosystems (Burlington, ON, Canada). Cultures were prepared from a frozen stock of N-38 neuronal cells and maintained in DMEM supplemented with 4.5 mg/ml glucose, 10% FBS, 1% penicillin/streptomycin, 0.15% sodium bicarbonate at 37°C, 5% CO_2_. Cells were plated in T75 flasks at 1,000,000 cells/flask 16 h prior to transfections.

### Real-Time PCR

Total RNA was isolated using TRIzol reagent (Life Technologies; Carlsbad, CA), according to the manufacturer’s protocol using 1 mL of TRIzol/100 mm plate. RNA from cells was extracted using chloroform. RNA pellets were washed with 100% isopropanol followed by 75% ethanol in DEPC-treated water. Pellets were allowed to dry for 10 minutes at room temperature and were resuspended in DEPC-treated water. RNA concentration and quality were assessed using a spectrophotometer (NanoDrop 1000, Thermo Fisher Scientific; Waltham, MA). 1–2 μg total RNA were used to synthesize cDNA with the SuperScript III Reverse Transcriptase kit (Invitrogen; Carlsbad, CA) using Oligo(dT)_20_ primers. The RT reaction was performed at 50°C for 50 minutes, followed by a 5 minute termination at 85°C. cDNA was used immediately for RT-PCR or stored at −20°C for ≤ 1 month.

Primers for neuropeptide Y and γ-actin were described previously [33]. Reactions were run on an Mx3000p thermal cycler (Agilent; Santa Clara, CA) using the program: 3 minutes at 95°C for DNA polymerase activation, then 40 cycles each consisting of 20 seconds at 95°C for denaturation and 20 seconds at 54°C annealing/extension temperature. Following amplification, a melting curve (54°C to 95°C) to identify PCR amplicons. In addition, PCR products were electrophoresed on a 2% agarose gel containing ethidium bromide (1.12 g agarose, Sigma #A9539; 50.4 mL dd H20; 5.6 mL 10x TAE buffer, Sigma #T8280; 1 uL EtBr) and visualized using the FluorChem E imager (ProteinSimple; Santa Clara, CA). A DNA ladder (GeneRuler 100 bp DNA ladder, Thermo Scientific) was run alongside samples for verification of amplicon size. All PCR products yielded single peaks in the melting curve analysis and single bands in agarose gel electrophoresis (S1 Fig.).

### siRNA Transfections

To knockdown the expression of Arrb1 in N-38 neurons, Arrb1 siRNA (cat. # SI02699116) and scrambled siRNA (cat. # 1027310) were purchased from Qiagen (Valencia, CA). Transient transfections were performed using Lipofectamine RNAiMax (Life Technologies, Grand Island, NY) with 50 nM scrambled or Arrb1 siRNA in OptiMEM reduced serum media (Life Technologies). Forty-eight hours after transfections, cells were steroid-starved for 20 h in charcoal-stripped media (phenol red-free DMEM supplemented with 5% charcoal-stripped/dextran-treated FBS (Gemini Bio-Products, West Sacramento, CA), 4.5 mg/ml glucose, 1% penicillin/streptomycin, 0.15% sodium bicarbonate) prior to biotinylation or internalization experiments.

### Surface biotinylation

Membrane ERα levels in the cell membrane of N-38 neurons were analyzed using a biotinylation procedure as described by Bondar, et al. [18]. Forty-eight hours after siRNA transfections, cells were treated with charcoal-stripped media (see above) for 16 h at 37°C, 5% CO_2_. Cells were treated with 1 nM water-soluble E2 (cyclodextrin-encapsulated; cat. #E4389, Sigma-Aldrich, St. Louis, MO). Following control or E2 treatment, cells were washed 3 times with ice-cold HBSS buffer and incubated with freshly prepared 0.5 mg/ml of cell-impermeable, non-cleavable EZ-Link Sulfo-NHS-LC-Biotin (ThermoFisher; Waltham, MA) in HBSS at 4°C for 30 min with gentle agitation. The biotin solution was aspirated and excess biotin was quenched by rinsing cells 3 times with ice-cold glycine buffer (50 mM glycine in HBSS). Cells were harvested in 15 ml HBSS and centrifuged at 850xg for 5 min at 4°C. Cell pellets were resuspended in 250 μL RIPA lysis buffer containing the following protease inhibitors: 1 mM phenylmethylsulfonyl fluoride, 1 μg/ml peptstatin, 1 μg/ml leupeptin, 1μg/ml aprotinin, 1 mM sodium orthovanadate (Santa Cruz Biotechnology, Santa Cruz, CA), and Halt phosphatase inhibitor cocktail (ThermoFisher). Cells were homogenized by passage through a 25 gauge needle. Lysates were clarified by centrifugation at 14,000xg for 5 min and collecting the supernatant. Protein concentration of the supernatant was determined using the BCA method (ThermoFisher). 200 μL of each sample with a protein concentration of 1500 μg/ml was incubated with 200 μl of immobilized NeutrAvidin beads (ThermoFisher) for 2 h at room temperature on an orbital shaker, followed by centrifugation for 1 min at 1000xg to separate proteins bound to the beads. The beads were washed 4 times with RIPA lysis buffer (containing the protease and phosphatase inhibitors listed above) before eluting bound proteins with Laemmli buffer (Bio-Rad, Hercules, CA) containing β-mercaptoethanol for 1 h at 37°C.

To study internalization of mERαΔ4, N-38 cells were biotinylated with cell-impermeable, SH-cleavable EZ-Link Sulfo-NHS-SS-Biotin (ThermoFisher; 0.5 mg/ml in HBSS) for 30 min at 4°C. After biotinylation and quenching, cells were treated with 1 nM E2 for 30 min before washing with chilled HBSS. Biotinylated proteins from cell surface were twice stripped with a membrane impermeant stripping buffer (100 mM mercaptoethanesulfonic acid [MESNA; Sigma-Aldrich], 50 mM Tris, 100 mM NaCl, 1 mM EDTA and 0.2% BSA, adjusted to pH 8.0). Cells were washed with 50 mM iodoacetamide to quench the MESNA. Internalization protected the biotin-labeled proteins from MENSA stripping. Preparation of cell extracts and biotinylated proteins were isolated as described above.

### Antibodies

A rabbit polyclonal antibody to the COOH-terminal of ERα was used (c1355; 1:1000; EMD Millipore, Billerica, MA) to detect ERα/ERαΔ4. For Arrb1, we used a rabbit antibody raised against the amino acid sequence of the NH_2_-terminal of Arrb1 (E274; 1:1000; Abcam). A rabbit polyclonal antibody directed against a synthetic phosphopeptide to residues surrounding Thr202/Thr204 of human p44 MAPK was used to detect dual phosphorylated-ERK1/2 (p44/42) (9101; 1:1000 Cell Signaling Technology, Danvers, MA). For total ERK1/2 (p44/42), a rabbit polyclonal antibody directed against the COOH terminus was used (9102; 1:1000; Cell Signaling Technology). GAPDH was used as a loading control and was detected with a mouse monoclonal antibody (6C5; 1:10,000; EMD Millipore). Secondary antibodies were horseradish peroxidase-conjugated goat-anti-rabbit (sc-2030; 1:10,000; Santa Cruz Biotechnology) or goat-anti-mouse (sc-2005; 1:10,000; Santa Cruz Biotechnology).

### Western Blots

Biotinylated membranes or cytoplasmic proteins were loaded onto 10% SDS polyacrylamide gels (Bio-Rad) and transferred to polyvinylidene difluoride (PVDF) membranes (GE Healthcare, Pittsburgh, PA). Nonspecific binding sites were blocked with 5% nonfat milk in TBS-T (Tris-buffered saline with 0.1% Tween 20) for 1 h on an orbital shaker. Primary antibodies were diluted in 5% bovine serum album (ERα, phospho-ERK1/2, or total ERK1/2) or 5% w/v nonfat milk (Arrb1, GAPDH) in TBS-T and incubated overnight at 4°C. Blots (PVDF membranes) were washed three times with TBS-T prior to incubation with secondary antibody (diluted in 5% nonfat milk in TBS-T) for 1 h. Blots were washed three times with TBS-T and once with TBS prior to visualization on a Fluor ChemE imager (ProteinSimple, San Jose, CA) using enhanced chemiluminescence (ECL; Western C, Bio-Rad).

### Densitometric analysis

Digitized images from a Fluor ChemE imager were analyzed using AlphaView 2.0 software (ProteinSimple). Total band intensity expressed as arbitrary optical density units for each sample were calculated by subtracting the background from each target signal. Phospho-ERK1/2 (phospho-p44/42) loading was normalized with total ERK1/2 to determine the amount of phosphorylated ERK1/2 protein. Variations in protein loading were corrected with GAPDH. Optical density ratios were normalized by dividing each set by the control ratio for each of the data sets and multiplied by 100 to obtain the relative change in optical density compared to the control group.

### Ethics Statement

This study was carried out in strict in accordance with the principles and procedures of the National Institute of Health *Guide for the Care and Use of Laboratory Animals*. The protocol was approved by the Chancellor’s Animal Research Committee at the University of California, Los Angeles (Protocol Number: ARC # 1999-020). All surgery was performed under isoflurane anesthesia, and all efforts were made to minimize suffering.

### Animals

Male and ovariectomized (ovx) female (200–250 g) Long-Evans rats were purchased from Charles River (Wilmington, MA). Upon arrival, rats were housed two per cage in a climate-controlled room, with a 12-h light, 12-h dark cycle (lights on at 0600h). Food and water were available *ad libitum*.

Bilateral guide cannulae (22 gauge; Plastics One Inc., Roanoke, VA) directed at the ARH (coordinates from bregma; −2.8 mm, lateral 0.8 mm, ventral −7.4 mm from dura; tooth bar: −3.3 mm) were implanted using standard stereotaxic procedures while female rats were anesthetized with isoflurane (2–3% in equal parts oxygen and nitrous oxide). Cannulae were secured to the skull with dental acrylic and stainless steel bone screws. Stylets were placed in the guide cannulae, which extended less than 0.5 mm beyond the opening of the guide cannulae. Animals were individually housed after surgery, received banamine (500 μg/0.1 ml s.c. injection every 12 h; Phoenix Pharmaceuticals, St. Joeseph, MO) and oral antibiotics (trimethoprim and sulfamethoxazole, 0.4 mg/ml; Hi-Tech Pharmacal, Amityville, NY) in the drinking water and were allowed to recover 6–7 days before steroid priming.

17β-estradiol benzoate (EB) dissolved in safflower oil was injected (s.c.) in a volume of 0.1 ml per rat. Females received 5 μg EB every 4 days between 0800h and 0900h for three cycles to mimic the natural estrous cycle of female rats as previously described [34].

### Microinjection

We compared the ability of siRNA and antisense oligodeoxynucleotides (asODN) to knockdown Arrb1 *in vivo*. The asODN produced greater knockdown of Arrb1 thus, this method was used to knock down Arrb1 *in vivo*. The asODN or nonsense scrambled oligodeoxynucleotides (nsODN) were dissolved in artificial cerebrospinal fluid for a final concentration of 3 μg/μl for the initial microinfusion and 2 μg/μl for the remaining microinfusions. The asODN cocktail consisted of three phosphorothiorated ODN targeted to the mRNA translation initiation site of Arrb1: 5’- *GTG*TCCCTTTGTCGC*C* *CA*T-3’, 5’-*ACA*CTCGTGTCCCTT*TGT*C-3’, and 5’- *TCC*CTTTGTCGCCCAT *GGT*C-3’. Phosphorothiorated nucleotides are indicated in italics and translation start site is underlined. The control, nsODN sequences were 5’- *CAC*AGGGAAACAGCG*GGT*A-3’, 5-*TGT*GAGCACAGGGAA*ACA*G-3’, and 5’- *AGG*GAAACAGCGGGTA*CCA*G-3’. A BLAST search revealed that none of the control nsODNs were predicted to bind any known rodent mRNA target. The ARH was microinfused bilaterally with 1μl at a rate of 0.25 μl/min using an infusion pump (Harvard Apparatus, Holiston, MA). Microinjection needles (28 gauge, Plastics One Inc) protruded 1 mm or less beyond the opening of the cannula and remained in place for 1 min after infusion to allow for diffusion away from the injector. After microinjection, obturators were reinserted into guide cannulae and animals were returned to their home cage.

To ensure a significant knockdown of Arrb1, animals received four microinfusions, one each consecutive day starting 24 h after the second EB injection. Microinfusions were 24 h apart. The final microinfusion was 30 min prior to the third EB injection, (i.e., 30.5 h before testing for lordosis behavior).

### Behavioral testing

Sexually experienced males were acclimated to Plexiglass arenas for 15 min before testing. Thirty hours after the third EB injection, to test for sexual receptivity, females were placed into an arena with a stud male. Males were allowed to mount females 10 times, and the number of times the female displayed lordosis (lifting of the head, arching of the back, and diversion of the tail to one side) was recorded. Sexual receptivity for each female was quantified as a lordosis quotient (LQ), defined as the number of lordosis displays/number of mounts x 100 (e.g., [2, 32, 35]).

### Confirmation of guide cannulae placement

Cannulae positions were visually confirmed during dissection. Rats with cannulae not positioned in the ARH (e.g., located dorsally or laterally to ARH, or if microinjections had compromised wall of the third ventricle, or the ventral surface of the brain) were excluded from the study. ARH tissue from animals with correct cannulae placements were homogenized in RIPA buffer and lysates were used for western blots to confirm Arrb1 protein knockdown.

### Statistics

Data are presented as means ± standard error (SEM) of a percent relative ratio. Statistical comparisons between 2 independent groups were made using the unpaired Student’s t-test. When comparing 3 or more independent groups, we used a one-way analysis of variance (ANOVA), with Student-Newman-Keuls post hoc test where appropriate. Data were analyzed using StatView (Version 5.0; SAS Institute Inc., Cary, NC).

## Results

**Fig 1:**
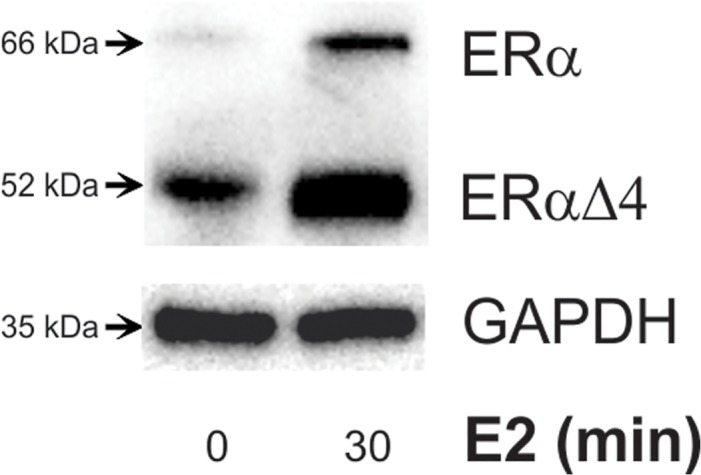
Cell surface biotinyation of N-38 cells treated with estrogen vs. control. Western blot analysis of biotinylated protein were probed with ERα to show: the 66 kDa band corresponding to the full-length ERα protein, and the 52 kDa band corresponding to a splice variant of ERα lacking exon 4 (ERαΔ4). The 52 kDa band is the primary ERα band detected in biotinylation/internalization experiments. Since both bands are regulated in parallel, ERαΔ4 was used as an indicator for both isoforms.

**Fig 2:**
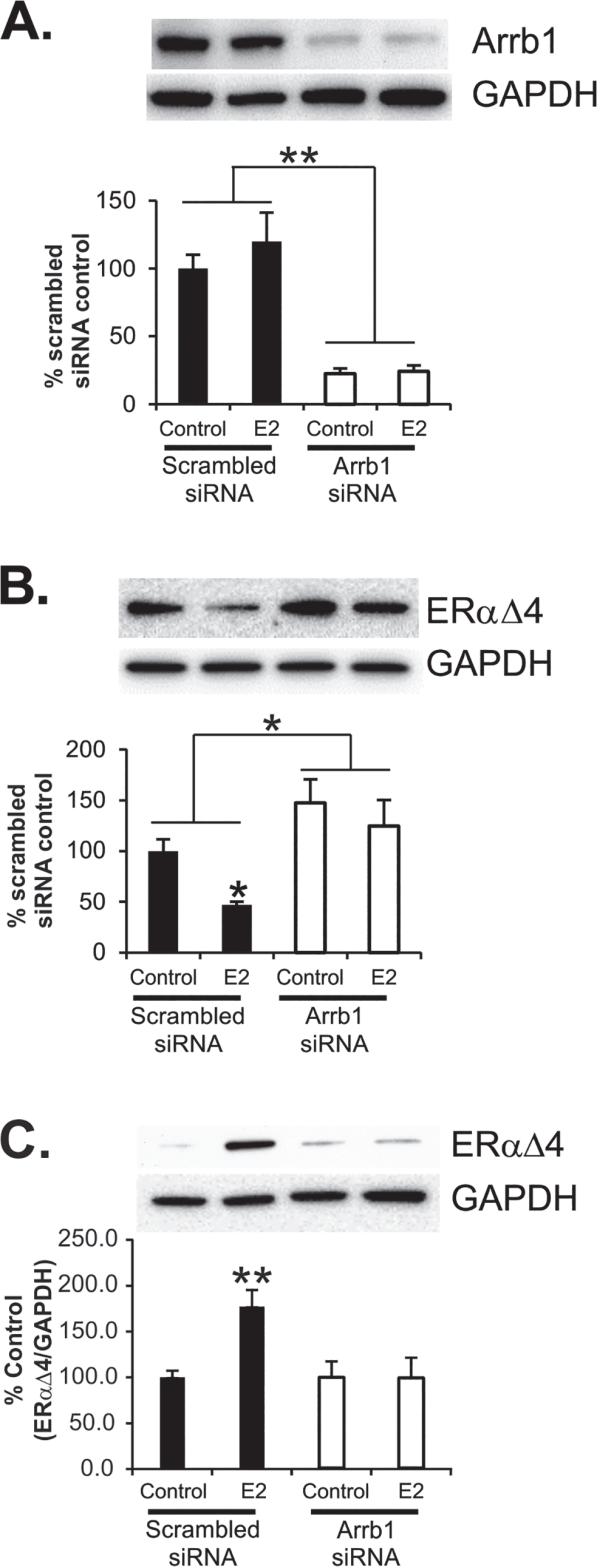
N-38 cell surface protein biotinylation and internalization after transfection with scrambled siRNA (control) or Arrb1 siRNA and estradiol (E2) treatment. (A) Arrb1 protein levels in N-38 cells transfected with scrambled siRNA (50 nM) vs. Arrb1 siRNA (50 nM) at 72 h post-transfection. (B) Cell surface mERαΔ4 levels in scrambled siRNA-transfected vs. Arrb1 siRNA-transfected cells in unstimulated cells or cells treated with E2 (1 nM for 2 h). (C) Internalization of mERαΔ4 in scrambled siRNA-transfected cells treated with E2 for 30 min vs. Arrb1 siRNA-transfected cells stimulated with E2. Values represent means (n = 6–7 experiments) ± SEM. *(p<0.05). **(p<0.005).

**Fig 3:**
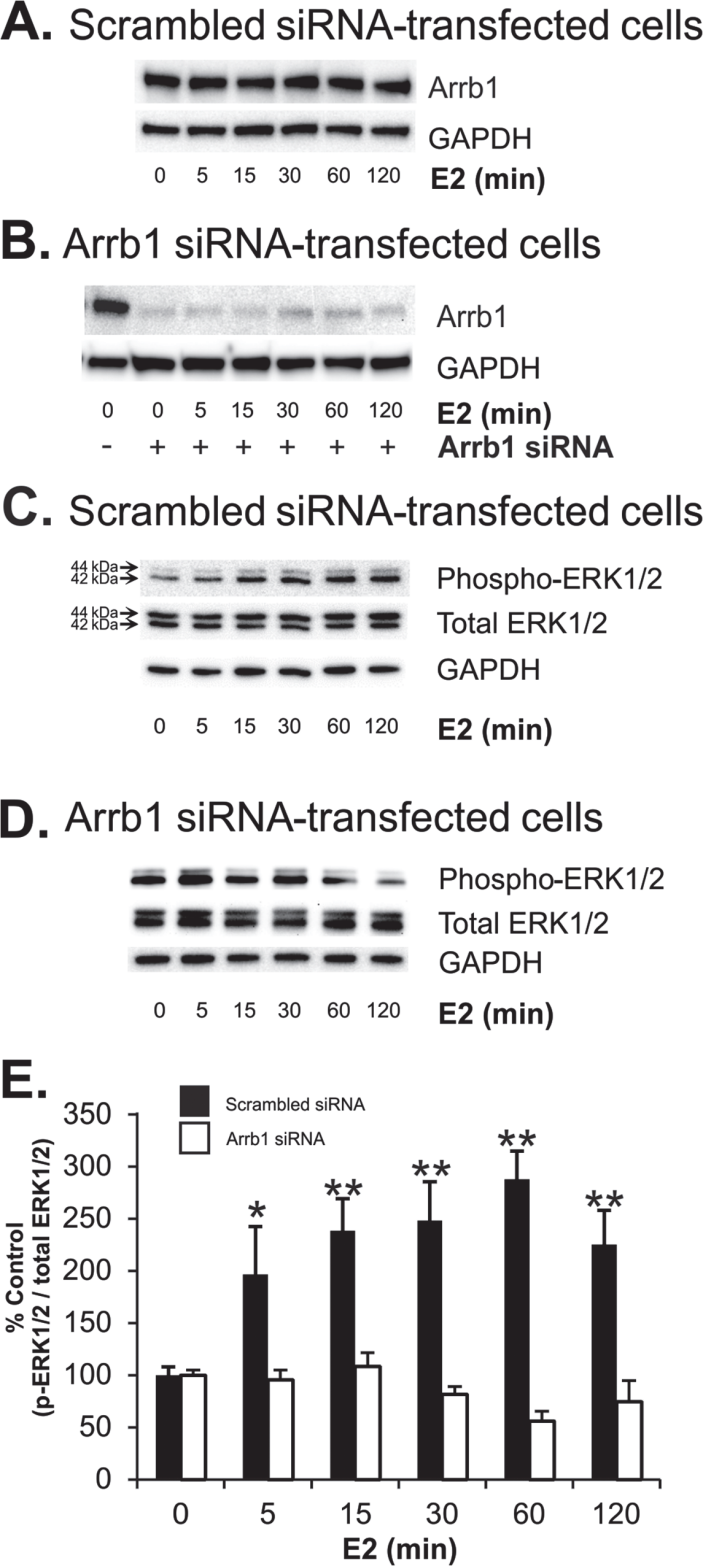
Estradiol (E2)-induced ERK1/2 phosphorylation in N-38 cells was attenuated in cells transfected with Arrb1 siRNA. (A) Western blots from scrambled siRNA-transfected N-38 cells probed with Arrb1 and GAPDH. (B) Arrb1 western blots from scrambled siRNA-transfected N-38 cells showing knockdown of Arrb1 protein. (C) Representative western blots of levels of ERK1/2 (p44/42 MAPK) phosphorylation in scrambled siRNA-transfected N-38 cells and (D) N-38 cells transfected with Arrb1 siRNA treated with estradiol (E2) for 0–120 min. (E) Densitometric analysis of the optical density from western blots of phosphorylated ERK1/2 (p44/42) protein bands normalized to the optical densities of total ERK1/2 protein in scrambled vs. Arrb1 siRNA-transfected cells. Controls for each group (cells not treated with E2) was set at 100%, and E2 treatments were expressed as a percentage of the control. Values represent means (n = 5) ± SEM. * (p<0.05). **(p<0.005).

To verify that N-38 neurons expressed NPY mRNA, quantitative PCR with published primer sequences [33] produced a 75 base pair amplicon (S1 Fig.). As we had previously reported, N-38s expressed ERα and ERαΔ4 in membranes (Fig. 1, [17]). Both isoforms were increased in parallel following a 30 min treatment with 1 nM E2 (Fig. 1, [17]). The predominant mERα variant in cultured hypothalamic cells (neurons and astrocytes) including N-38s is the 52 kDa ERαΔ4 (Fig. 1, [16–18]). ERαΔ4 is coded for by an alternatively spliced ERα mRNA lacking exon 4 of ESR1 and is found throughout the brain [19]. As reported in previous studies, ERαΔ4 is found in the cell membrane *in vivo* and *in vitro*, but its levels are higher than full length ERα *in vitro* where both the full-length and the ERαΔ4 are regulated in parallel (Fig. 1, [16–18]). In this study, full-length ERα was barely detectable following surface biotinylation, so ERαΔ4 was used as a marker of cell surface membrane ERα levels (Figs. 2 and 3).

### Arrb1 protein knockdown increases membrane ERαΔ4 in N-38 cells

Levels of Arrb1 were not sensitive to E2 treatment, however, transfection of N-38 neurons with Arrb1 siRNA significantly reduced levels of the protein in both control and E2-treated cultures by 80% [Fig. 2A; t(34) = -7.296, *p<*0.0001]. Treatment with E2 (1 nM) reduced cell surface membrane levels of ERαΔ4 by 53% [Fig. 2B; scrambled siRNA vehicle: 100.0 ± 11.7 vs. E2 treated 46.9 ± 3.4%, F(3,23) = 6.147, *p<*0.005]. However, in Arrb1 siRNA-transfected N-38s, membrane levels of ERαΔ4 were significantly increased compared with scrambled siRNA-transfected cells [Fig. 2B; t(25) = 3.373, *p<*0.005], suggesting that internalization was affected, but not trafficking to the membrane. Arrb1 knockdown prevented E2-induced internalization of mERαΔ4 [Fig. 2B; Arrb1 siRNA vehicle: 147.5 ± 23.2, vs. Arrb1 siRNA E2: 124.9 ± 25.5, F(3,23) = 6.147, *p =* 0.384], and this was highlighted by the difference between mERαΔ4 levels in scrambled siRNA/E2-treated versus Arrb1 siRNA/E2 treated N-38 cells, suggesting that Arrb1 mediated mERαΔ4 internalization [Fig. 2B; scrambled siRNA E2: 46.9 ± 3.4 vs. Arrb1 siRNA E2: 124.9 ± 25.5, F(3,23) = 6.147, *p*< 0.01].

### Arrb1 protein knockdown attenuates membrane ERα internalization

To confirm that Arrb1 knockdown prevented mERαΔ4 internalization, N-38 neurons were surface biotinylated with an SH-cleavable biotin, treated with E2 and then stripped of surface biotin with MESNA. Only proteins that were internalized remained biotinylated because they were not exposed to MESNA. The biotinylated proteins were concentrated using an avidin column. Thirty min E2 treatments increased internalized mERαΔ4 levels following transfection with scrambled siRNA compared with non-E2 treated cells [Fig. 2C; 30 min E2: 177.3 ± 18.2 vs. Vehicle (no E2): 100.0 ± 7.3%; t(12) = −3.937, *p*<0.005]. In contrast, Arrb1 siRNA knockdown inhibited E2-induced mERαΔ4 internalization [Fig. 2C; 30 min E2: 99.4 ± 22.1vs. Control: 100.0 ± 18.1%; t(12) = 53, *p* = 1.000]. These results demonstrated a critical role for Arrb1 in mediating E2-induced mERαΔ4 internalization.

### E2-mediated ERK1/2 (MAPK) activation depends on Arrb1

Since Arrb1 knockdown impaired mERαΔ4, and by extension ERα, internalization, intracellular signaling was examined to determine the consequence of inhibiting internalization. Arrb1 protein levels were significantly reduced in Arrb1 siRNA-transfected cells compared to scrambled siRNA-transfected cells [Fig. 3A and 3B]. In N-38 neurons transfected with scrambled siRNA, E2 treatment induced a rapid 2-fold increase of phosphorylated ERK1/2 (phospho-p44/42) levels. The increase was detected within 5 min of E2 treatment and remained elevated for up to 2 h [Fig. 3C and 3E; 5 min E2: 196.4 ± 46.2, 15 min E2: 238.4 ± 30.7, 30 min E2: 248.2 ± 37.1, 60 min E2: 287.8 ± 26.9, 120 min E2: 225.1 ± 32.9% vs. Control: 100.0 ± 8.0%; F(5,43) = 6.667, *p*<0.0005]. In Arrb1 siRNA-transfected N-38 cells, E2 did not induce ERK1/2 phosphorylation [Fig. 3D and 3E; 5 min E2: 95.7 ± 9.4, 15 min E2: 108.4 ± 13.2, 30 min E2: 81.6 ± 7.5, 60 min E2: 56.0 ± 14.8, 120 min E2: 74.7 ± 20.1% vs. Control: 100.0 ± 5.1%; F(5,36) = 1.832, *p* = 0.1313]. These data indicate that Arrb1 is involved in EMS downstream signaling.

### Arrb1 knockdown attenuates EB-induced lordosis behavior

**Fig 4:**
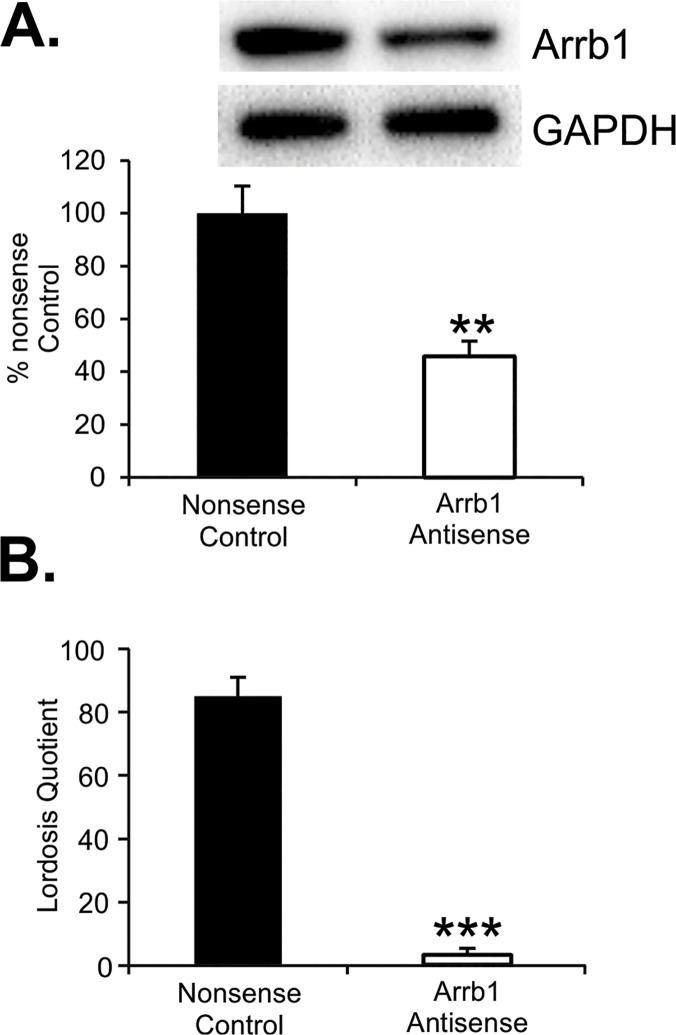
Arrb1 mediates EB-induced lordosis behavior. *In vivo*, Arrb1 asODN significantly reduced levels of Arrb1 in the ARH of ovariectomized, EB-treated rats. (A) Arrb1 protein levels in ARH lysates from females microinjected on four consecutive days with Arrb1 asODN versus females microinjected with nsODNs. (B) Lordosis quotient from rats injected with nsODNs vs. Arrb1 asODNs. Values represent means (n = 8–10 animals) ± SEM. ** (p<0.005). ***(p<0.0001).

The ARH is the critical site for EMS modulation of sexual receptivity [2, 10]. The initial E2 action stimulates NPY neurons that in turn activate an opioid circuit, whose transient inhibition is necessary for the full expression of lordosis behavior 30–48 h after EB treatment of ovariectomized rats [36]. To test whether Arrb1 is involved in lordosis, an EMS-mediated behavior, we knocked down Arrb1 protein in the ARH using Arrb1 asODN. *In vivo*, we found that Arrb1 knockdown was more robust with Arrb1 asODN compared with Arrb1 siRNA (data not shown); therefore asODN were used in the behavioral experiments. Microinjecton of Arrb1 asODN reduced Arrb1 protein by 55% compared with control nsODNs [Fig. 4A; asODN: 45.8 ± 5.7% vs nsODN: 100.0 ± 10.3; t(15) = −4.853, *p*<0.0005]. In females microinjected with Arrb1 asODN, lordosis quotients were severely attenuated 31 h after EB treatment [Fig. 4B; nsODNs: 85.0 ± 6.0 vs. asODN: 3 ± 2.1; t(15) = 14.086, *p*<0.0001]. Although sexually proceptive behavior was not quantified, females treated with Arrb1 asODN displayed less ear wiggling, hopping and darting than nsODN or stimulus females (intact females treated with 50 μg EB). These results are consistent with the idea that EMS *in vivo* requires Arrb1.

## Discussion

The major findings of the present study are that Arrb1 participates in downstream signaling initiated by estradiol action at the membrane, including the activation of ERK1/2, which continued even after receptor internalization. Importantly, Arrb1 expression in the ARH was necessary for lordosis behavior, a finding consistent with the proposed role of Arrb1 in EMS. These results underscore the importance of EMS as a mode of E2 activation of physiological processes including sexual receptivity. Our results support the idea that Arrb1 is a scaffolding protein organizing downstream signaling molecules and as an adaptor protein aiding in ER internalization after its activation. This is the first indication that membrane-initiated estradiol signaling involves Arrb1, although such a role has been described for other membrane receptors [24–26, 37].

N-38 neurons are a viable model in which to examine EMS. These immortalized hypothalamic cells express NPY, ERα and ERαΔ4. E2 increases [Ca^2+^]_i_, and activates PKCθ in these neurons. The present study confirms that trafficking of full-length ERα and the ERαΔ4 are regulated in parallel by E2, as they are in primary cultures of hypothalamic neurons and astrocytes [16–18, 38].

Interestingly, ERαΔ4 is enriched in the membranes of primary cultures or are cell lines derived from neurons and astrocytes [16–18, 38]. However, in tissue from the ARH, full length ERα, not ERαΔ4, was the predominant ERα isoform on the cell membrane, [35]. In that study, we found that caveolin-1 which is necessary for ERα trafficking to the membrane did not affect membrane ERαΔ4 levels suggesting another scaffolding molecule is needed to traffic this isoform. At this point, we do not understand the conditions that shift the proportion of ERαΔ4 to ERα in cultured cells compared with tissue. Its presence in membrane fractions is likely due to the deletion of exon 4, which results in an in-frame deletion of ERα that gives rise to a truncated protein [39–41]. The missing exon codes for the nuclear localization sequence and part of the ligand-binding domain [16–18, 42, 43]. There is little consensus about the nature of ERαΔ4 signaling or transcriptional activity. Since ERαΔ4 lacks the nuclear localization sequence, it has been hypothesized that after translation ERαΔ4 would not be transported back to the nucleus. This hypothesis was support in ERαΔ4-overexpressing COS7 cells in which ERαΔ4 was localized only in the cytoplasm [44]. However, in another study, overexpression of rat pituitary ERαΔ4 in COS-1 cells the isoform was detected in both cytosol and nucleoplasm [43]. Functionally, ERαΔ4 was initially thought to be a silent variant since ERαΔ4 expression in JEG3 placental choriocarcinoma **cells did** not stimulate transcription [45]. Importantly, it did not bind E2 or interact with the estrogen response element (ERE), and had no dominant negative effects on full-length ERα activity. Similar effects were found when rat ERαΔ4 was expressed in A10 smooth muscle cells [46]. In another study, human ovarian ERαΔ4 was overexpressed in COS-1 cells [47]. ERαΔ4 did not activate transcription but did inhibit full-length ERα transcriptional activation via protein-protein interaction. Despite these results, when rat pituitary ERαΔ4 was overexpressed in Chinese hamster ovary (CHO) cells and GH4C1 rat pituitary cells, ERαΔ4 modulated transcription [43]. Interestingly, this transcriptional activity was found in the absence of E2. Further, phosphorylated-ERαΔ4 was also found to bind a consensus ERE.

Such differences may reflect cell-type specific co-activators/co-repressors involved in estrogen signaling. For example, other ERα splice variants had different transcriptional activity between HeLa cervical cancer and M17 neuroblastoma cells, which was attributed to the differential co-activators/co-repressers present in each cell type [48]. In our hands, EMS requires the interaction of mGluR1a with full length ERα [2, 3, 18] but these studies did not demonstrate, ERαΔ4-mGluR1a interactions suggesting that EMS is not mediated by ERαΔ4.

Thus, the function of ERαΔ4 is not known at present. ERαΔ4 was first described in breast cancer cell lines and meningiomas [39, 41]. The lack of transcriptional activity and dominant negative effects on full-length ERα suggested that ERαΔ4 was a silent splice variant that played no role in tumor progression [45]. In support of this, levels of ERαΔ4 mRNA did not differ between normal and breast cancer tissues [49]. Significantly, ERαΔ4 has also been detected in normal brain tissue, bone and vascular smooth muscle cells, but its function in these tissue is also not characterized [40, 46, 50]. One possibility is that ERαΔ4 may have a role in development since full-length ERα and ERαΔ4 were found to be differentially regulated during rat pituitary development [51]. We do not know whether this is the case in the brain. In present study, we did not examine the developmental regulation of ERαΔ4. Moreover, in all our in vitro studies, E2 regulated both full-length ERα and ERαΔ4 in parallel. Therefore, we have used ERαΔ4 as a marker for full-length ERα, whose expression is lower *in vitro*.

**Fig 5:**
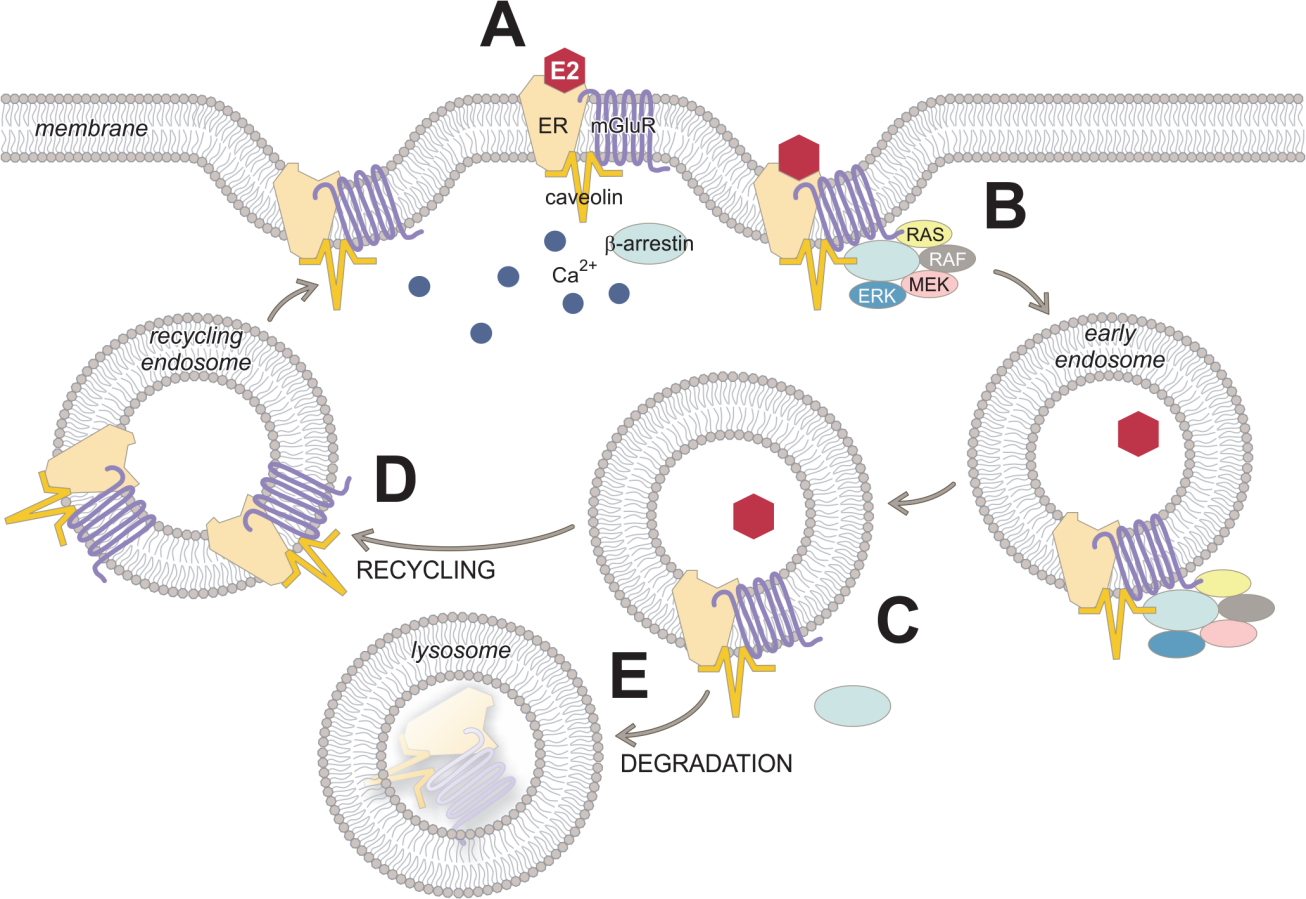
Proposed mechanism for the role of Arrb1 in internalization and signaling of mERα/ ERαΔ4 (depicted as mERα in figure for simplicity). (A) Membrane ERα is part of a G-protein coupled receptor complex which includes mGluR1a and caveolin (reviewed in [5]). (B) Following E2 activation of mERα, Arrb1 is recruited to this receptor complex where it binds organizes Raf/MEK/ERK signaling and the endocytic machinery needed to internalize mERα into endosomes. In the absence of Arrb1, ERα internalization and ERK1/2 (MAPK) signaling are blocked. Eventually, the internalized mERα-mGluR1a loses Arrb1 (C), signaling ceases and the receptor complex is (D) recycled and trafficked to the cell surface, or (E) sorted to lysosomes for degradation.

Previously, we reported that full-length ERα interacts with mGluR1a to induce downstream signaling (Fig. 5; reviewed in [36]). Activated mERα is internalized through a mechanism involving phosphorylation by GRK2, recruitment of Arrb1 and internalization of the receptor complex [15]. While we used ERαΔ4 as a surrogate for full-length ERα, the E2 signaling we see in N-38s is dependent on mGluR1a and by extension ERα. The data are consistent with the idea that Arrb1 links mERα and ERαΔ4 to the AP-2 adaptor complex to assist clathrin-mediated endocytosis, a traditional role for β-arrestins [23, 37, 52].

Internalization is important for the immediate reduction of receptors on the cell surface which can lead to a cessation of signaling or the recycling of ligand-free receptors now available for restimulation [18]. Once in early endosomes, receptors release their ligands and can be trafficked back to the membrane restoring cellular responsiveness. Thus, the rapid internalization and trafficking back to the cell surface may aid in cellular responsiveness by returning receptors to the plasma membrane. Alternatively, internalized receptors can be sorted to lysosomes where they are proteolytically degraded leading to a down regulation of receptors and a prolonged attenuation of cellular responsiveness. Rapid trafficking to the cell surface and internalization followed by down regulation have been described for mERα in cells of the nervous system [16, 18]. While we do not entirely understand the process controlling the fate of internalized receptors (recycling versus degradation), the present studies indicate that Arrb1 plays a central role in the initial internalization of the receptor.

Both G protein and Arrb1 can mediate ERK1/2 activation [53–55]. The present results indicate that Arrb1 is directly involved in EMS potentially by serving as a scaffold to recruit and organize downstream signaling molecules (e.g., Ras/Raf/MEK) at the cell membrane [24, 26, 56, 57]. Moreover, Arrb1 has been implicated as a mechanism through which endosomal signaling extends cellular responsiveness (reviewed in [58, 59]). One distinction is that G protein-specific-mediated ERK1/2 activation is relatively rapid (maximal levels 2–5 min post-stimulus) and transient with a duration of approximately 60 min [53, 60, 61], whereas β-arrestin-mediated phosphorylation persists longer (>90 min) [26, 53, 55, 61, 62]. Our results are most consistent with β-arrestin-mediated signaling. E2 induced ERK1/2 phosphorylation in 5 mins, and it persisted longer than G protein-only mediated signaling (∼2 h). Furthermore, Arrb1 siRNA inhibited ERK1/2 activation throughout the time course of the experiment (5 to 120 mins). Phosphorylation sites in the GPCR COOH-terminus contribute to the stability of the GPCR-arrestin-ERK1/2 complex, and affect the subcellular location and function of activated ERK1/2 such that stable GPCR-arrestin complexes are associated with prolonged endocytic signaling and ERK1/2 signaling [62–65]. E2-induced ERK1/2 phosphorylation and internalization were both increased within 5 min and were both disrupted by Arrb1 siRNA, consistent with the idea that Arrb1 is involved in the initial GPCR signaling and its prolongation. This suggests that EMS persists as long as Arrb1 remains associated with the receptor, including after sequestration into endosomes [65].

A physiological consequence of Arrb1 knockdown was assessed by examining lordosis, a female sexual receptivity reflex we had previously shown to be dependent on EMS in the ARH [10, 35, 66–68]. ERα transactivation of mGluR1a in the ARH activates PKC, leading to rapid μ-opioid receptor activation within the medial preoptic nucleus. This transient inhibition is necessary to facilitate lordosis 30–48 h post-EB treatment [2, 10]. Antagonizing ERα, mGluR1a or PKC in the ARH at the time of EB treatments attenuates lordosis. More recently, we found that E2, PKC and CAV1 regulate ERα trafficking to the membrane, which is necessary for inhibiting ERα-dependent lordosis behavior [17, 68]. Similarly, in the present experiments, female rats receiving Arrb1 asODN into the ARH were unreceptive showing almost no lordosis behavior. The present results may provide a mechanism that underlies activation of female sexual receptivity with E2 without progesterone. Large doses of EB (50 μg) induce lordosis within 48 hours while more physiological doses (2 μg) do not (e.g., [69]). Lower doses of EB maintain EMS, resulting in the inhibition of lordosis. In the present study, low E2 doses prolong Arrb1-mediated activation of EMS, which would extend inhibition of lordosis. In gonadally intact rats, EMS-mediated inhibition of lordosis is relieved by progesterone [32].

An important consideration is that the neural circuitry regulating lordosis behavior relies on E2 stimulation of ERα in ARH neurons. Arrb1 mediates cell signaling in a large number of GPCRs and so we cannot be certain that the effect of Arrb1 knockdown on lordosis behavior was specific to disruption of EMS. At present we cannot rule out that other receptor signaling was also affected by Arrb1 knockdown producing an effect on lordosis but through a different mechanism. Our Arrb1 asODN treated animals did not show reduced locomotion, signs of sickness, weakness or changes in appetite suggesting that our treatments did not compromise general activity in the ARH.

In summary, the present results demonstrate the importance of Arrb1 in EMS and regulation of mER levels on the membrane. In immortalized hypothalamic NPY neurons, Arrb1 knockdown attenuated E2-induced mERαΔ4 internalization and phosphorylation of ERK1/2. EMS is part of E2-mediated events in the nervous system that include nociception, addiction and plasticity [70–74]. In terms of reproduction, perhaps the most studied E2 action in the CNS, EMS is important for both the regulation of the luteinizing hormone (LH) surge in estrogen positive feedback and the regulation of sexual receptivity [36, 67]. In the present experiments, we demonstrated that Arrb1-mediated EMS was critical for E2-induced lordosis behavior. E2-transactivation of the GPCR, mGluR1a, initiates cell signaling pathways (e.g., ERK1/2) necessary for spinogenesis and the activation of the lordosis-regulating circuitry [66]. Fundamentally, mERα is regulated like other membrane receptors. These studies extend our understanding of mERα trafficking, signaling and internalization. Since EMS continues after ERα is sequestered into early endosomes these studies identify an additional level of complexity for estrogenic effects in the nervous system.

## Supporting Information

S1 Fig PCR data with neuropeptide Y (NPY) and γ-actin primers on cDNA from N-38 cells. (A) SYBR-green dissociation curves from NPY and γ-actin amplicons. (B) Image of NPY and γ-actin amplification products run on a 2% agarose gel with ethidium bromide.
(TIF)
