# Supplementary figures and images for: β-Arrestin Regulates Estradiol Membrane-Initiated Signaling in Hypothalamic Neurons
Source: PLoS One. 2015 Mar 24;10(3):e0120530. doi: 10.1371/journal.pone.0120530 (PMC4372564; doi:10.1371/journal.pone.0120530)

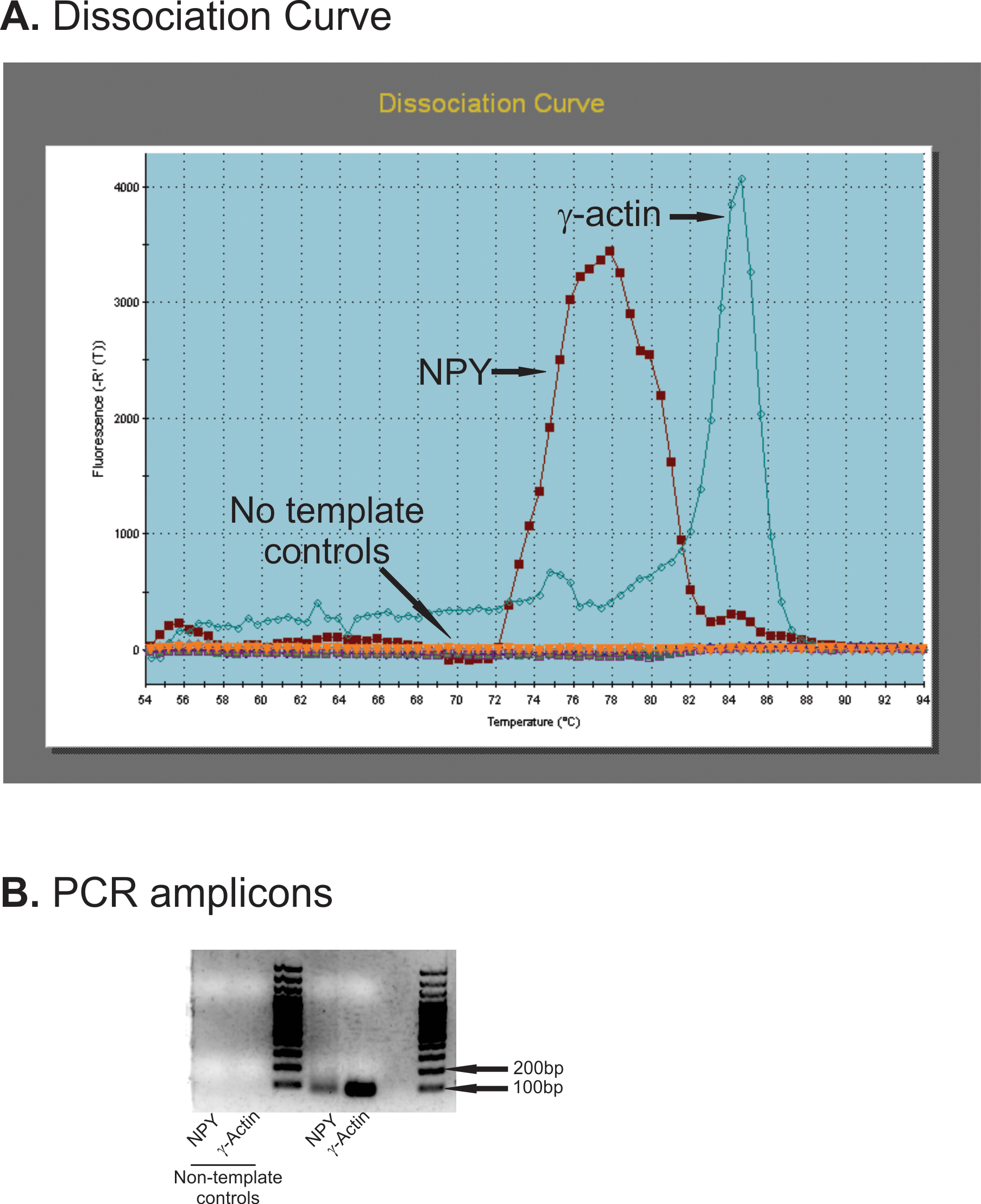

Supplement: S1 Fig — (A) SYBR-green dissociation curves from NPY and γ-actin amplicons. (B) Image of NPY and γ-actin amplification products run on a 2% agarose gel with ethidium bromide. (TIF) [file pone.0120530.s001.tif]
